# Topics of Public Interest

**Published:** 1911-04

**Authors:** 


### TOPICS OF PUBLIC INTEREST

### Micro-organism Found in the Blood of Acute Cases of Poliomyelitis—Department of Health, Pennsylvania

IN examining the blood from acute cases of poliomyelitis in the human beings and also in monkeys in which the disease was produced experimentally an organism was found, different in morphologic characteristics from any heretofore described which may or may not, on further investigation, prove to be the etiological factor in the causation of the disease. Blood smears being fixed in methyl alcohol for one minute and stained with carbolthionin, the organism appears as a faintly stained blue rod with regular cell wall about 10 microns long and about .8 microns in width, curved at an angle of sixty to seventy-five degrees at one end, occasionally at both ends. At times, the curved end is bulbous. Some of the organisms appear to have a very finely granular protoplasm when the highest amplification is employed. They may be discerned by means of a 4 m.m. dry objective but their characteristics are much more satisfactorily delineated under the 1-12 oil immersion lens. They are found free in the serum as well as within the body of the red blood cell.

The organisms do not retain the violet color when stained by the method of Gram but assume the color of the counter stain which, as generally used in this laboratory, is a very dilute solution of carbol fuchsin.

The bloods examined were from ten different cases of acute poliomyelitis in children and were taken during the epidemic of last summer and autumn, and from thirteen cases of the disease during the acute stage, which had been produced experimentally in as many monkeys. Blood smears from three normal human beings were carefully examined and although the search for these organisms was diligently made, none were found. Smears were made from the bloods of thirteen normal monkeys with negative results. After inoculation with the virus these same monkeys gave positive results. The blood of other normal monkeys gave negative results. Blood smears were stained with iodine and sulphuric acid in order to test the organisms for cellulose, but no blue stained organisms were seen. Smears from the cords and brains of paralyzed monkeys, and from one human case were examined, but none of the new organisms were found.

Filtered virus stained with carbol-thionin and by Gram's method showed none of these organisms. Defibrinated blood, three weeks to two months old from two paralyzed monkeys showed the forms in increased numbers. Cultures made from the blood of a paralyzed monkey, in blood bouillon, plain bouillon, and blood agar, examined after having been inoculated three weeks, showed the presence of the organism in increased numbers. Dorsett's egg medium was inoculated with the same blood at the same time but the organism was not found in smears from the surface of the medium or from the water of condensation.

Searches have been made without success for moving organisms in fresh blood, in old tubes of defibrinated blood from paralyzed monkeys, in blood bouillon, plain bouillon, serum bouillon cultures three weeks old and in the condensation water in three weeks old cultures on Dorsett's egg medium under dark field illumination. The efforts in isolating the organisms have not, as yet, been successful.

